# Nanovesicles and Human Skin Interaction: A Comparative Ex-Vivo Study

**DOI:** 10.3390/nano15120937

**Published:** 2025-06-16

**Authors:** Elisabetta Esposito, Valentyn Dzyhovski, Federico Santamaria, Catia Contado, Cinzia Brenna, Luca Maria Neri, Paola Secchiero, Francesco Spinozzi, Alessia Pepe, Michał Rawski, Maria Grazia Ortore, Paolo Mariani, Andrea Galvan, Laura Calderan, Manuela Malatesta

**Affiliations:** 1 Department of Chemical, Pharmaceutical and Agricultural Sciences, University of Ferrara, I-44121 Ferrara, Italy; dzyvnt@unife.it (V.D.); federico.santamaria@edu.unife.it (F.S.); kat@unife.it (C.C.); 2 Laboratory for Technologies of Advanced Therapies “LTTA”-Electron Microscopy Center, University of Ferrara, I-44121 Ferrara, Italy; cinzia.brenna@unife.it (C.B.); luca.neri@unife.it (L.M.N.); 3 Department of Translational Medicine and for Romagna, University of Ferrara, I-44121 Ferrara, Italy; paola.secchiero@unife.it; 4 Department of Life and Environmental Sciences, Marche Polytechnic University, I-60131 Ancona, Italy; f.spinozzi@univpm.it (F.S.); a.pepe@univpm.it (A.P.); m.g.ortore@univpm.it (M.G.O.); p.mariani@univpm.it (P.M.); 5 SOLARIS, National Synchrotron Radiation Centre, Jagiellonian University, 30-392 Kraków, Poland; michal.rawski@uj.edu.pl; 6 Department of Neurosciences, Biomedicine and Movement Sciences, University of Verona, I-37134 Verona, Italy; andrea.galvan@univr.it (A.G.); laura.calderan@univr.it (L.C.); manuela.malatesta@univr.it (M.M.)

**Keywords:** skin, ethosome, liposome, transmission electron microscopy, hyperspectral dark microscopy, microfluidics

## Abstract

The topical administration of drugs on the skin by nanovesicular systems can represent a tool to treat skin pathologies. The study of nanovesicle biodistribution after skin administration is crucial to understanding their transdermal potential. A formative study enabled us to investigate the influence of some methods in the production of nanovesicles based on phosphatidylcholine, differing in their ethanol amount. Particularly, both liposomes and ethosomes produced by different methods, i.e., microfluidics and solvent injection, were considered. The evaluation of size distribution, shape and internal morphology was performed using photon correlation spectroscopy, cryogenic electron microscopy, hyperspectral dark-field microscopy and small-angle X-ray scattering. Transmission electron microscopy was then used to observe and compare the transdermal passage of selected liposomes and ethosomes applied to human skin explants in a bioreactor. The mean diameters of nanovesicles prepared by the ethanol injection method were smaller with respect to those obtained by microfluidics, measuring roughly 140 and 230 nm, respectively. The uni- or multilamellar ultrastructure of the vesicles was influenced by the solvent injection procedure. Ultrastructural analysis of skin penetration revealed (i) the ability of intact vesicles to cross the different skin layers, with ethosomes produced by the water injection method showing greater transdermal potential and (ii) the role of ethanol as a penetration enhancer.

## 1. Introduction

Skin’s primary function is to protect the body against exogenous stimuli, such as insults, infections, as well as exposure to environmental stressors [1]. The protective effect is mainly exerted by the *stratum corneum*, acting as the major barrier. Although the skin offers a potential route for drug administration, especially topically for skin conditions, the *stratum corneum* presence hinders drug passage. Consequently, in recent years, significant research has been focused on developing effective transdermal drug delivery systems to overcome this barrier [2,3,4]. Particularly, liposomes (LIPO) and ethosomes (ETHO) are colloidal delivery systems made of biocompatible and biodegradable phospholipids organized as bi-layered vesicles dispersed in water [4,5,6,7,8,9]. Typically, the main component of LIPO and ETHO is represented by phosphatidylcholine (PC), a zwitterionic surfactant that spontaneously self-aggregates in water, giving rise to lamellar structures [10]. The ultrastructural organization of LIPO and ETHO allows the solubilization of both hydrophilic and lipophilic compounds, promoting their transdermal delivery. Indeed, due to their affinity with the skin architecture and composition, LIPO and ETHO endorse the active compound passage through the upper skin strata towards the dermis [4,5,6,11]. LIPO and ETHO have proven effective in biomedical applications, improving the delivery of therapeutics for various skin disorders. ETHO formulations show promise for treating viral (e.g., acyclovir for herpes) [12], fungal (e.g., fluconazole) [13], and inflammatory (e.g., colchicine for psoriasis) skin conditions [14]. These carriers facilitate drug penetration into deeper skin layers, enhancing treatment outcomes for conditions like acne, psoriasis, and skin cancers. For instance, tretinoin ETHO offers improved efficacy and reduced irritation compared to conventional acne treatments [15]. Initially utilized in dermatology for their moisturizing and regenerative effects, the focus of LIPO and ETHO research has shifted towards their ability to encapsulate and deliver biological materials to epidermal cells and deeper skin layers [16,17].

The main difference between LIPO and ETHO lies in the ethanol presence, potentially reaching 45% *w*/*w* in ETHO. Ethanol plays diverse pivotal roles; indeed, on the one hand, it makes the vesicles more flexible; on the other, it interacts with skin and vesicle lipids, helping the drug associated with the vesicles to pass through the *stratum corneum* [18]. Additionally, ethanol noticeably improves vesicle stability and its ability to hold lipophilic drugs [19,20,21,22]. Some authors demonstrated that LIPO exhibited insufficient flexibility for skin penetration, resulting in the formation of a drug depot within the *stratum corneum*, from which encapsulated drugs diffused slowly [23]. Consequently, deformable LIPO was engineered through the incorporation of edge activators (e.g., surfactants), thereby destabilizing the lipid bilayers composed of PC and water and, in turn, increasing vesicle flexibility [24,25]. Analogously, the addition of edge activators to ETHO resulted in transethosomes in an attempt to further increase the vesicle penetration potential [22,26].

Various techniques have been created over time to yield lipid-based nanocarriers. Most of these “top-down” methods rely on physically breaking down larger, pre-formed structures. Sonication is a key example, though its scalability is limited. Conversely, “bottom-up” approaches offer an alternative by promoting the self-assembly of lipids in solution into lipid-based nanocarriers, thus avoiding the need for size reduction [27].

Different “bottom-up” methodologies can be employed for LIPO and ETHO preparation [28]: (i) the classic thin-film hydration method, based on the hydration of a thin lipid film obtained under evaporation of an organic solvent from a lipid solution; (ii) ethanol injection, involving the injection of a lipid ethanol solution into water; (iii) reverse-phase evaporation, forming LIPO by creating a water-in-oil emulsion from which the organic solvent is evaporated, leading to LIPO formation; and (iv) microfluidics, a more modern approach based on the use of microchannels to mix lipid solutions and aqueous phase [28].

The choice of method depends on the desired LIPO size, lamellarity (number of lipid bilayers), and the type of drug to be encapsulated. Post-processing steps, such as size reduction and purification, may be necessary to obtain the desired vesicle features [29]. Proper characterization of nanovesicles for skin application is essential for a complete understanding of their behaviors on the skin and their biological effects [30]. Key physical attributes that significantly impact skin interactions, such as size distribution, shape, and inner morphology, should be thoroughly investigated [31].

A crucial point in the potential of a transdermal delivery system is related to the maintenance of its structural integrity throughout the skin strata to carry the associated drug and release it deeply toward the dermis.

Despite several in vitro and in vivo studies have examined the transdermal effects of LIPO and ETHO, focusing on drug delivery and therapeutic potential [22,26,32,33], a limited number of studies have been dedicated to investigating the effective cutaneous penetration of LIPO and ETHO when applied onto the skin [34,35].

Recently, we investigated ETHO and transethosomes applied on healthy human skin explants maintained in a bioreactor, an in vitro model that reliably simulates in vivo physiological conditions and preserves skin structure for extended periods [36]. Visualization of vesicles within skin strata was accomplished through transmission electron microscopy (TEM) experiments, enabling them to unequivocally determine their penetration potential and their fate in the different skin layers. The mean size, morphology, and deformability of these vesicles were shown to be affected by polysorbate 80, used as an edge activator in transethosome production, and by the concentration of PC [36].

In the present work, a comparative study was carried out, comparing the LIPO and ETHO penetration capability throughout healthy human skin.

LIPO and ETHO produced by different methodologies, such as microfluidics, water injection, and ethanol injection, were characterized and compared for their physical-chemical features, such as size distribution, determined by photon correlation spectroscopy (PCS), and morphology, evaluated by cryogenic electron microscopy (Cryo-EM), Small Angle X-Ray Scattering (SAXS) and hyperspectral dark-field microscopy [37].

In the second part of the study, the transdermal passage of selected LIPO and ETHO applied on human skin explants maintained in a bioreactor was monitored and compared by TEM [38]. TEM allows for direct visualization of nanovesicles within the skin, shedding light on the impact of methodology on nanovesicle physicochemical features and cutaneous fate [39]. 

## 2. Materials and Methods

### 2.1. Materials

Phosphatidylcholine (PC) (purity 94%) from soybean was acquired by A.C.E.F. Spa. (Milan, Italy). The solvents used were HPLC grade, while all other chemicals were analytical grade.

### 2.2. Preparation of Nanovesicular Systems

LIPO and ETHO production was made alternatively by a microfluidic approach or by two bulk methods, specifically “water injection” and “ethanol injection”.

#### 2.2.1. Microfluidic Approach

For the microfluidic approach, the preparation of LIPO_MF_ and ETHO_MF_ was performed using a cross-junction microfluidic chip based on a Large Droplet Junction Chip (Dolomite, Alfatest, Rome, Italy) with a hydrophilic quartz channel etched to a depth of 100 µm, mounted on a Chip interface H equipped with two Linear Connector 4-way (Dolomite, Alfatest, Rome, Italy).

The inner lipid phase (LP) consisted of a PC ethanol solution (30 mg/mL), while bi-distilled water served as the aqueous outer phase (AP). The AP and LP flow rates (F_AP_ and F_LP,_ respectively) were regulated using two syringe pumps (IPS-14 syringe series, Inovenso Inc., Başakşehir/Istanbul, Turkey). Visualization was performed with an optical microscope (Leica DM LS2, Leica Microsystems Srl, Buccinasco (MI), Italy). The Flow Rate Ratio (FRR) between F_LP_ and F_AP_ was set to 10:1 and 2:1 *v*/*v*, respectively for LIPO_MF_ or ETHO_MF_. The Total Flow Rates (TFR) were 55 and 90 µL min^−1^.

After stabilization of the focused stream, each sample (3 mL) was collected into glass vials and stored at 4 °C; sample preparation was performed at 25 °C.

#### 2.2.2. Bulk Approach

In the case of the bulk approach, LIPO and ETHO preparation was performed by a cold method alternatively based on the dropwise addition of bidistilled water to PC ethanol solution (“water injection”) or by the dropwise addition of PC ethanol solution to bidistilled water (“ethanol injection”).

Particularly, in the case of the water injection method, LIPO_WI_ and ETHO_WI_ were prepared dripping bidistilled water at 1500 μL min^−1^ (IPS-14 syringe series, Inovenso Inc., Başakşehir/Istanbul, Turkey) in the PC ethanol solution (30 mg/mL) kept under magnetic stirring at 750 rpm (IKA RCT basic, IKA^®^-Werke GmbH and Co. KG, Staufen, Germany). The final water/ethanol ratios were 90:10 (*v*/*v*) for LIPO_WI_ and 70:30 for ETHO_WI_.

For the ethanol injection method, LIPO_EI_ and ETHO_EI_ were prepared by adding the PC ethanol solution (30 mg/mL) to bidistilled water dropwise, maintaining the same flow, stirring rates and time requirements as for the water injection method. The final water/ethanol ratio was 90:10 or 70:30 (*v*/*v*) for LIPO_EI_ and ETHO_EI,_ respectively.

In both the water injection and ethanol injection methods, stirring was carried out at 22–25 °C for 30 min after the water or ethanol phases had been added.

### 2.3. Vesicle Characterization

#### 2.3.1. Photon Correlation Spectroscopy (PCS)

Vesicle size distribution was determined using a Zetasizer Nano-S90 (Malvern Instruments, Malvern, UK) equipped with a 5 mW helium-neon laser operating at a wavelength of 633 nm. Measurements were conducted at 25 °C, using a detection angle of 173°, following a 120-s equilibration period. Prior to analysis, samples were diluted 1:10 (*v*/*v*) with bidistilled water. The size distribution was calculated using the CONTIN algorithm [40]. All measurements were performed in triplicate, and the results are reported as mean values.

Moreover, the size distribution of samples stored at 22–25 °C for 3 months was measured to gain information on vesicle size stability.

#### 2.3.2. Cryogenic Electron Microscopy (Cryo-EM)

Cryo-EM data were acquired at the National Cryo-EM Centre SOLARIS in Kraków, Poland, using a Titan Krios G3i transmission electron microscope (Thermo Fisher Scientific, Brainport Eindhoven, The Netherlands) operated at 300 kV. Images were recorded at a nominal magnification of ×105,000, corresponding to a calibrated pixel size of 1.7 Å, using EPU 2.10 software. A K3 direct electron detector (Gatan, Pleasanton, CA, USA), operated in counting mode and integrated with a BioQuantum Imaging Filter (20 eV slit width) (Gatan, Pleasanton, CA, USA), was employed for data collection. The total electron dose applied was 41.09 e^−^/Å^2^, with a defocus setting of −2.0 µm.

Prior to imaging, samples were plunge-frozen in liquid ethane using a Vitrobot Mark IV (Thermo Fisher Scientific, Brainport Eindhoven, The Netherlands). For each grid, 3 µL of the sample suspension was applied, and excess liquid was blotted from both sides. Vitrification was performed under controlled conditions: 95% humidity, 4 °C temperature, 3 s blot time, and blot force setting of 3.

#### 2.3.3. Small Angle X-Ray Scattering (SAXS)

SAXS measurements were performed at the Austro-SAXS beamline of the Elettra Synchrotron facility in Trieste, Italy [41]. The samples were enclosed in quartz capillaries with an inner diameter of 1.5 mm and maintained at a constant temperature of 32 °C using a temperature-controlled sample holder equipped with Peltier heating/cooling (KPR, Anton Paar, Graz, Austria).

For each measurement, the sample was illuminated with an X-ray beam for 10 s per frame, and a total of 18 frames were acquired. The raw two-dimensional scattering images were processed to correct for background noise, detector sensitivity, and transmission effects. Following these corrections, the data were azimuthally averaged to obtain the one-dimensional scattering profile, expressed as the differential scattering cross-section $\frac{d\Sigma }{d\Omega }$ or the intensity I(Q), where Q represents the magnitude of the scattering vector and is calculated as Q = 4π sin(θ)/λ (with 2θ being the scattering angle and λ the wavelength of the incident X-rays).

The sample-to-detector distance was set to a Q-range of approximately 0.1 to 5 nm^−^¹. Scattering patterns were recorded using a two-dimensional Pilatus3 1M detector, equipped with a pixel size of 172 × 172 μm^2^. The detector operated at a photon energy of 8 keV, corresponding to an X-ray wavelength of 0.154 nm.

The uncertainties on all fitting parameters were estimated using the GENFIT software, version 2024.11 (available at https://sites.google.com/site/genfitweb/download) by repeating the fitting procedure 20 times. In each repetition, the experimental SAXS data points were randomly perturbed by sampling from a normal distribution with a standard deviation equal to the experimental error associated with each q-point. The mean value and standard deviation of each parameter were then calculated from the 20 values obtained across the repeated fits.

#### 2.3.4. Hyperspectral Dark-Field Microscopy

Five microliters of LIPO_EI_, ETHO_WI_, or ETHO _EI_ dispersion was placed on a glass slide (Epredia Glass Slides 76 × 26 mm, provided by Menzel Gläser, Geelong, Australia) and let dry until the solvent fully evaporation to prevent light scattering phenomena. Before imaging, a coverslip was placed on the sample. Hyperspectral imaging was performed using a CytoViva^®^ dark-field hyperspectral microscopy system (CytoViva Inc., Auburn, AL, USA), which included a halogen light source, a BX-43 research-grade optical microscope, and a hyperspectral imaging setup. The system was equipped with a sCMOS spectral camera coupled to a VNIR imaging spectrograph (ImSpector V10E, Specim, Spectral Imaging Ltd. Oulu, Finland), enabling data acquisition across the visible to near-infrared (VNIR) range of 400–1000 nm.

Acquisition parameters were: (i) light intensity of 80% of maximum, (ii) 100× immersion oil objective (Olympus UPlan FL N, 100×/1.30), (iii) spatial resolution (x;y) of 1024 × 1024, (iv) spectral resolution (λ) of 1.2 nm, (v) exposure time 0.2 s.

Post-process imaging was performed with ENVI 4.0 (CytoViva^®^ proprietary software): a minimum of 3 regions of interest (ROIs) or 15 single pixels, located in different areas of the image and characterized by their light intensity value 10% below the maximum level, were selected to obtain specific and representative spectra.

### 2.4. Human Skin Sampling

Skin samples were surgically removed from the abdominal region of four healthy women undergoing abdominoplasty at the Verona University Hospital. The patients signed an informed consent. The study was conducted in accordance with the Declaration of Helsinki and authorized by the Institutional Ethics Committee of the Verona University Hospital (protocol code 3449CESC, 21 April 2021). The excised skin samples were immediately washed in physiological solution (NaCl 0.9% *w*/*v*), placed in Dulbecco’s Modified Eagle’s Medium (DMEM), supplemented with 4.5 g/L D-glucose, 10% FBS, 2% penicillin-streptomycin, 200 mM L-glutamine, and 0.3 μg/mL Amphotericin B (Gibco, Waltham, MA, USA) at 37 °C for a few minutes, and then processed for treatment in the bioreactor.

### 2.5. Skin Treatment and Transmission Electron Microscopy (TEM) Analysis

**Figure 1:**
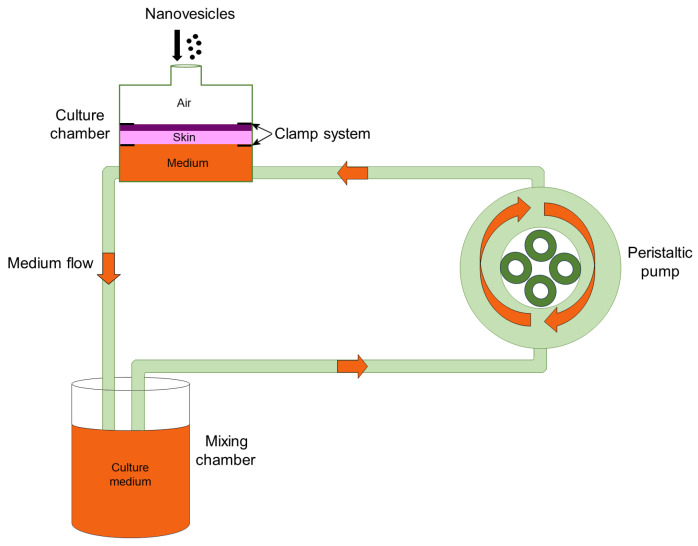
Schematic representation of the bioreactor used in this study.

The bioreactor used in this study (IV-Tech, Massarosa, LU, Italy) is schematically represented in Figure 1 and has been extensively described in [38].

Round samples with a diameter of 1.5 cm were placed in culture chambers modified to house flat organs (LiveBox2; IV-Tech): the epidermis was placed at the top, in contact with air, while the dermis was placed at the bottom, in contact with the flowing (500 μL/min) culture medium [38]. A 300 µL drop of LIPO_EI_, ETHO_WI_, or ETHO_EI_ dispersion (PC 0.9% *w*/*w*) was applied to the *stratum corneum*. The bioreactor was maintained in an incubator at 37 °C in a 5% CO_2_ humidified atmosphere for 1 h, 3 h, 6 h, and 24 h. As a control, some skin samples were treated with the dispersion medium devoid of nanovesicles. After incubation, skin samples were aldehyde-fixed (2% (*v*/*v*) paraformaldehyde and 2.5% (*v*/*v*) glutaraldehyde in 0.1 M phosphate buffer) for 3 h at 4 °C, treated with 1% (*v*/*v*) OsO_4_ and 1.5% (*v*/*v*) C_6_FeK_4_N_6_ for 1.5 h, dehydrated with acetone and embedded in epoxy resin (Epon-Araldite, Electron Microscopy Sciences, Hatfield, PA, USA). Sections of 70–90 nm in thickness were placed on copper grids (Electron Microscopy Sciences) and stained with Reynold’s lead citrate. Observations were made with a Philips Morgagni transmission electron microscope (FEI Company Italia Srl, Milan, Italy) equipped with a Megaview II camera for digital image acquisition.

LIPO_EI_, ETHO_WI_, or ETHO_EI_ diameter was measured in TEM micrographs (×22,000) with the ImageJ software 1.10z (NIH), and the mean and standard deviation (s.d.) values were obtained (n = 20 for LIPO_EI_, ETHO_WI_, or ETHO_EI_). In the case of flattened nanovesicles, the major axis was considered.

## 3. Results

### 3.1. Preparation of Nanovesicles

Nanovesicles based on PC were designed as biocompatible nanocarriers for dermal and transdermal delivery of drugs. Both LIPO and ETHO were investigated, embodying in their composition different amounts of ethanol, respectively 10 or 30%, *w*/*w*. The presence of ethanol, acting as a penetration enhancer in conjunction with PC, allows the vesicles to reach deeper skin levels. A previous study on ETHO, conducted by our research group, led to the selection of a 0.9% *w*/*w* PC, resulting in stable vesicles with a mean diameter of around 200 nm and a homogeneous size distribution [42,43,44]. Moreover, PC concentration equal to 9 mg/mL is an intermediate between the typical lipid concentration range of 5 to 15 mg/mL in liposomal nanomedicines [45,46]. This concentration was achieved using initial PC solutions 90 and 30 mg/mL for LIPO and ETHO, respectively.

In the present investigation, LIPO and ETHO produced by different procedures were compared.

As the first approach, a microfluidic method was employed using a device equipped with two syringe pumps connected to a chip containing two intersecting microchannels [47]. Control was maintained using two flow regulators and an optical microscope. Within the microfluidic chip, the LP and AP flows converged at the intersection of the two channels. The subsequent diffusion of water and ethanol at their interface triggered the self-assembly of the PC, reproducibly generating vesicles of precise and controlled size [48].

As a second approach, two bulk methods were employed, namely “water injection,” (extensively studied by our group) based on adding water by a syringe pump to a PC solution in ethanol under magnetic stirring, and “ethanol injection” method, where PC ethanol solution is gradually added to bidistilled water, under stirring [49].

Table 1 summarizes the parameters and compositions employed for nanovesicle preparation. The TFR and FRR parameters were selected based on a previous study conducted by our research group [48] aimed at obtaining vesicles with homogeneous size distribution and mean diameter around 200 nm. Indeed, PCS’s previous experiments let us demonstrate that lower TFR resulted in smaller monodispersed vesicles (lower D.I.), while higher TFR led to larger and polydisperse vesicles. Also, a high TFR (around 90 µL/min) led to phase separation of the LIPO dispersions. This was attributed to the potential development of unstable flow patterns within the microchip, negatively affecting the mixing of AP and LP.

In the case of vesicles prepared by the bulk method, in both LIPO and ETHO, F_AP_ and F_LP_ were 1500 µL/min, thus extremely greater with respect to those employed for the microfluidics method.

In the case of vesicles prepared by microfluidics, TFR and FRR were adjusted to obtain different amounts of ethanol, namely 3-fold higher in the case of ETHO_MF_ compared to LIPO_MF_. In both LIPO and ETHO, ethanol enables to maintain the vesicle stability. Typically, the presence of 20–45% *w*/*w* ethanol differentiates ETHO from LIPO, resulting in more stable and softer vesicles while improving their penetration potential.

Nanovesicular systems appeared milky, translucent, and homogeneous, apart from LIPO_WI_ that, showed phase separation 1 day after preparation (Table 2).

### 3.2. Size Distribution

PCS enabled the measurement of the size distribution of vesicles one day after their preparation. Table 2 reports the main size distribution parameters as well as information about the macroscopic aspect of the different nanovesicular systems evaluating by visual inspection possible phase separation phenomena.

**Figure 2:**
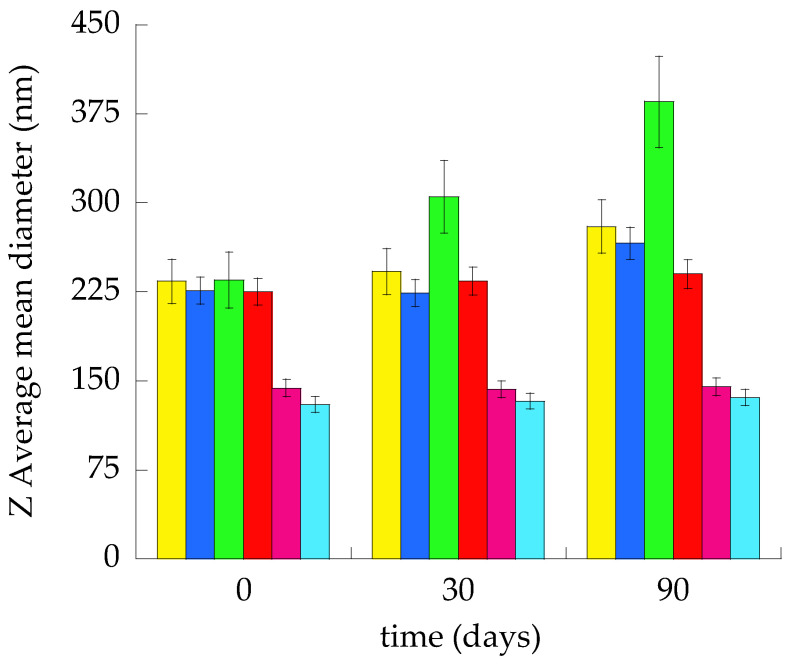
Variation of Z Average mean diameters of LIPO_MF_ (yellow), ETHO_MF_ (blue), LIPO_WI_ (green), ETHO_WI_ (red), LIPO_EI_ (violet), and ETHO_EI_ (light blue).

The average hydrodynamic diameters ranged between 130 and 235 nm, maintaining dispersity indexes always below or equal to 0.27. The smallest vesicles were obtained by the bulk ethanol injection method. Indeed, mean diameters were 70–100 nm smaller than the corresponding vesicles produced by the water injection method. In general, ETHO vesicles displayed slightly smaller mean diameters with respect to LIPO. It was found that nanovesicular systems produced by the ethanol injection method were characterized by Z-Average mean diameters smaller with respect to the ones obtained by the water injection method. Conversely, this latest method resulted in vesicles whose mean diameter value was near the vesicle’s mean diameter prepared by microfluidics. Since LIPO_WI_ displayed a macroscopic aspect different from other nanovesicular systems, a size stability study was performed, measuring Z Average mean diameters up to 3 months from the vesicle preparation (Figure 2).

As clearly detectable, the more stable nanovesicular systems were ETHO_EI_, ETHO_WI,_ and LIPO_EI_, maintaining almost unvaried Z-Average mean diameters with respect to the initial values (1–6%). Conversely, ETHO_MF_, LIPO_MF_, and LIPO_WI_ underwent a Z-average increase of 18, 20 and 64%, respectively.

The largest mean diameter and dispersity index detected after LIPO_WI_ preparation is responsible for vesicle aggregation and phase separation phenomena.

Because of size distribution, stability, and macroscopic aspect, LIPO_EI_, ETHO_WI_ and ETHO_EI_ were selected for further studies.

### 3.3. Morphological Characterization

**Figure 3:**
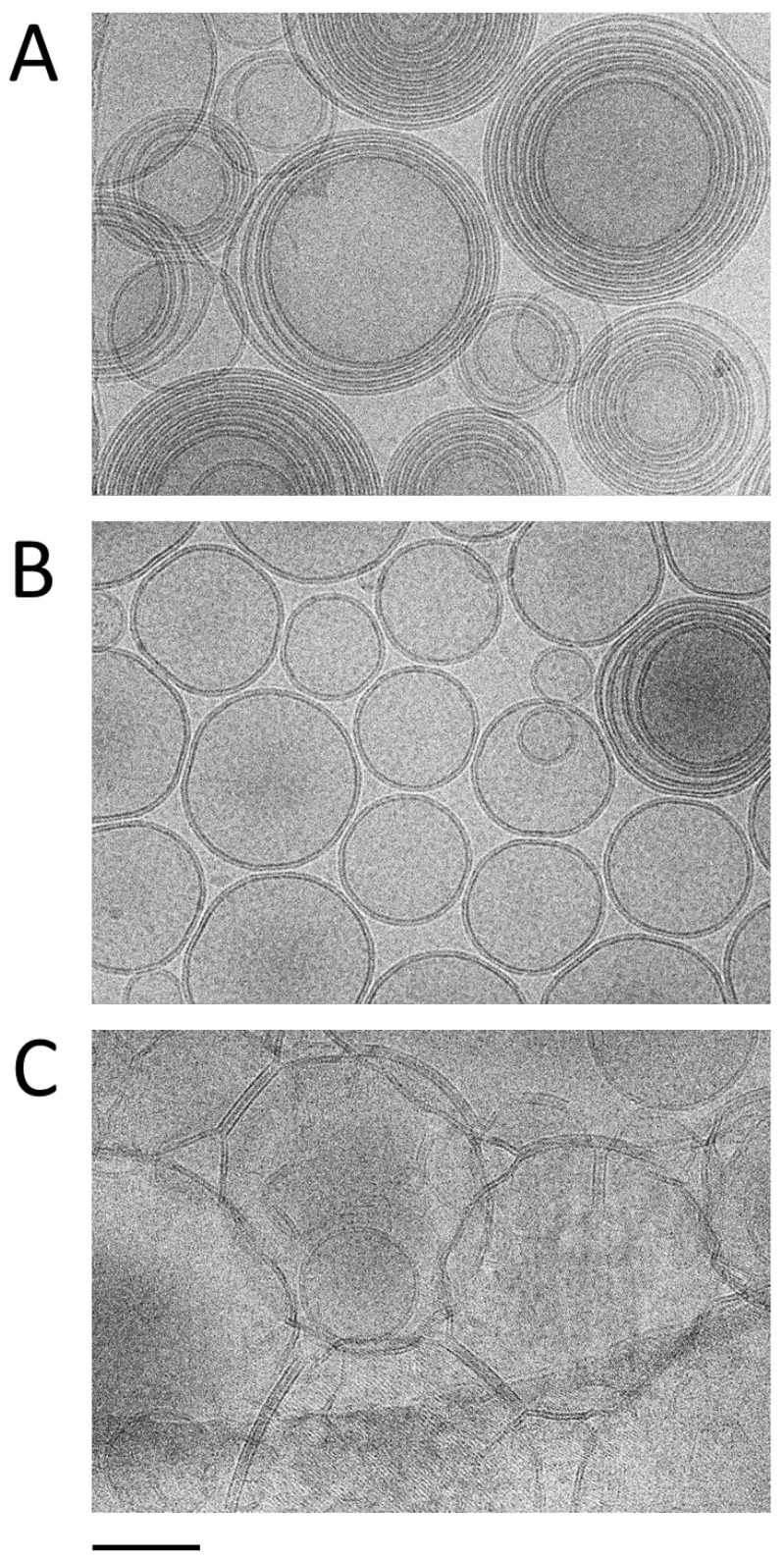
Cryo-EM images of ETHO_WI_ (**A**), ETHO_EI_ (**B**), and LIPO_EI_ (**C**). The bar corresponds to 100 nm.

The morphology of nanovesicles obtained via the ethanol and water injection methods was evaluated using Cryo-EM. The resulting micrographs corresponding to LIPO_EI_, ETHO_WI_ and ETHO_EI_ (Figure 3A–C) show spherical vesicles, whose size distribution is in agreement with PCS analysis, with a very well-defined double layer. Notably, the water injection method (Figure 3A) resulted in multilamellar vesicles, whilst the ethanol injection method (Figure 3B,C) yielded a predominance of unilamellar and oligolamellar vesicles, as previously observed in the case of gossypin-loaded ETHO [49]. In the case of LIPO_EI_ (Figure 3C), the vesicles appear more irregular and have a tendency to aggregate. These observations underscore the impact of the preparation technique on nanovesicle morphology. It can be hypothesized that, in the water injection method, the gradual addition of water to the PC-ethanol solution, on the one hand, facilitates a more controlled vesiculation process, leading to larger structures; on the other, it promotes the assembly of multiple lipid bilayers, resulting in the observed prevalence of multilamellar vesicles. Conversely, the rapid removal of the organic solvent during ethanol injection could accelerate vesiculation, thereby impeding the formation of complex multilamellar structures [49].

### 3.4. SAXS Study

**Figure 4:**
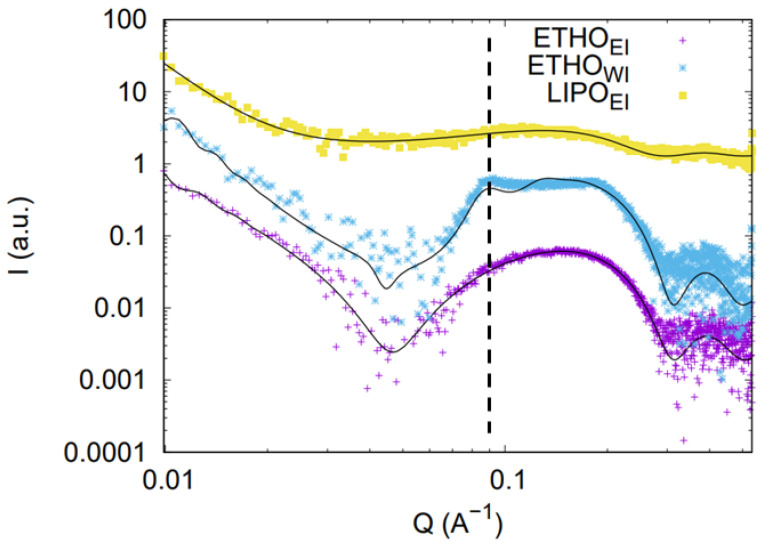
Comparison among the SAXS profiles of ETHO_WI_ (blue), ETHO_EI_ (purple), and LIPO_EI_ (yellow). The black vertical line indicates the position of the Bragg peak at Q = 0.087 Å^−1^ observed in the ETHO_WI_ profile. Model fitting curves are also reported.

SAXS was employed to investigate the structural characteristics of LIPO and ETHO produced via ethanol injection and water injection methodologies. Representative SAXS profiles, acquired at 32 °C, are presented in Figure 4. Although the data for LIPO show rather weak intensity, all curves exhibit a broad scattering band in the range of 0.6 to 3 nm^−1^, confirming the presence of the lipid bilayer in the ETHO_WI_, ETHO_EI_, and LIPO_EI_ vesicles, in good agreement with Cryo-EM results. A main distinction between the formulations is the appearance of a clear Bragg reflection at *Q* = 0.087 Å^−1^ in the ETHO_WI_ profile. This observation is interpreted as evidence of a prevalent multilamellar vesicle structure in this sample, thus validating the results obtained through Cryo-EM. Furthermore, the Bragg peak corresponds to an interlamellar distance of 7.22 nm, which aligns with previously reported data for comparable drug-delivery systems [43]. A final point to note is that the overall SAXS intensity for LIPO_EI_ is very low, likely due to a low scattering contrast between the components, which may be attributed to the higher water content inside the LIPO nanovesicles (90:10 compared to 70:30 in ETHO).

To derive quantitative information, SAXS profiles have been then fitted considering the nanovesicles as a polydisperse system of spheres with core radius *R_0_* and a dispersion ξ*_Ro_* described by a Schultz distribution [34]. The nanovesicle core is filled with a water/ethanol solution. Surrounding the core, there is a symmetric bilayer composed of three concentric shells with constant electron densities: the polar head group (thickness *R*_1_ and electron density *ρ*_1_), the central aliphatic chain region (thickness *R*_2_ and electron density *ρ*_2_), and the terminal methyl group (thickness *R*_3_ and electron density *ρ*_3_). Additional bilayers, identical in composition and structure to the one enveloping the nanovesicles and spaced by layers of water with electron density *ρ*_0_ can also be included in the model: each bilayer is defined by the same parameters *R_i_* and *ρ_i_* (*i* = 1, 2, 3). These *N* repeating bilayers are distributed with an average inter-bilayer spacing *c*, and their positional disorder is accounted for using paracrystalline theory with distortion parameter *g_c_*.

The fitting curves and corresponding best-fit parameters are shown in Figure 4 and summarized in Table 3.

Fitting data confirm the qualitative observations: in all cases, the fit indicates the presence of vesicles with dimensions of a few hundred nanometers and with lipid membranes of similar thickness (around 3.6 nm). However, nanovesicle structural characteristics depend both on composition and preparation method: ETHO_WI_ vesicles (with a low polydispersity in size) show a multilamellar structure with a low degree of cumulative positional disorder, whilst ETHO_EI_ vesicles (rather monodisperse in size) are predominantly unilamellar or oligolamellar. On the contrary, LIPO_EI_ vesicles show a strong degree of disorder with regard to the interplanar distances between the lamellae. Moreover, the fitting results are fully consistent with the suggested variation in scattering contrast between components due to the increased water content inside the LIPO nanovesicles.

### 3.5. Hyperspectral Dark-Field Microscopy Study

Hyperspectral Light Microscopy is a potent analytical technique for nanoparticulate and nanovesicular systems that integrates spectral data acquisition with optical microscopy methods like fluorescence, brightfield, or darkfield. This synergy enables the extraction of richer information about the sample being studied. The technique utilizes hyperspectral imaging sensors to capture UV/Vis or infrared spectra from closely spaced points, organizing the data into three-dimensional cubes. In these cubes, the x and y axes represent spatial information, while the z-axis corresponds to a specific range of wavelengths. The unique spectrum associated with each pixel allows for the accurate identification of observed objects, enabling precise analysis and mapping [50,51,52]. ROIs can also be visually defined to aggregate spectral data from numerous pixels, yielding an average spectrum for the selected area [52]. This average spectrum is constructed from the underlying spectra of the individual pixels within the ROIs. The combination of dark-field microscopy and Hyperspectral Imaging provides a powerful approach for spectrally characterizing materials with a far superior resolution than standard brightfield microscopy. Given that dark-field mode can image nanoparticles down to 5 nm, these particles can subsequently be analyzed using hyperspectral data acquisition. Specifically, hyperspectral analysis proves useful for identifying whether nanoparticles/nanovesicles are isolated or aggregated in aqueous environments and inside living cells.

**Figure 5:**
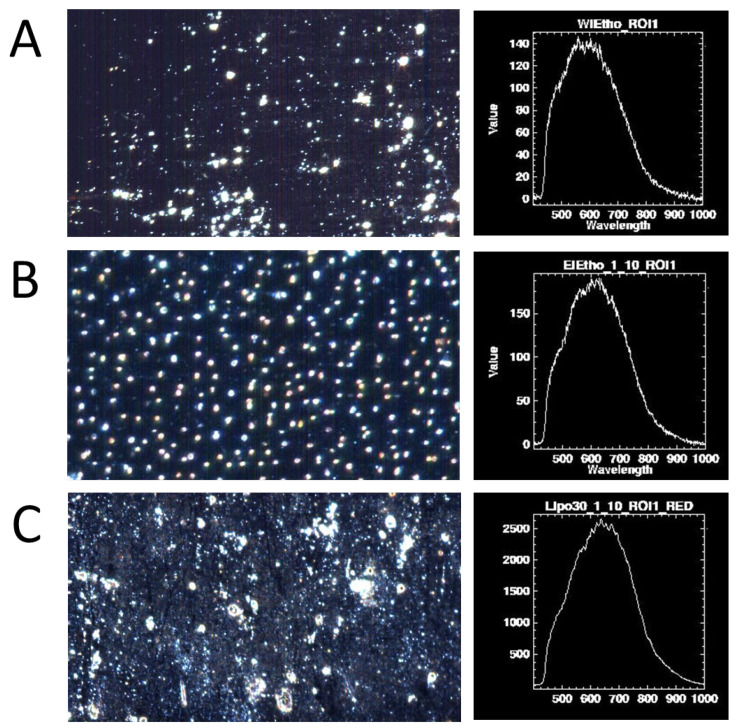
Hyperspectral images of ETHO_WI_ (**A**), ETHO_EI_ (**B**) and LIPO_EI_ (**C**) were acquired by enhanced dark-field microscopy using Cytoviva. In the right column, the corresponding spectra collected in isolated points are reported.

In Figure 5, hyperspectral images of the different nanovesicular systems are compared. In the case of ETHO_WI_ (Figure 5A) and ETHO_EI_ (Figure 5B), nanovesicles are individually dispersed, while LIPO_EI_ shows some aggregates (Figure 5C). This is in agreement with cryo-EM findings. In all cases, light scattering spectra show similar maximum intensities, indicating a uniform size distribution of the lipid vesicles.

### 3.6. Transmission Electron Microscopy Analysis of Skin Treated with ETHO and LIPO

ETHO_WI_, ETHO_EI_ and LIPO_EI_ were applied on healthy human skin explants maintained in a bioreactor able to improve structural and functional preservation [38]. This experimental procedure allowed us to track nanovesicles in the different skin strata at increasing incubation times, namely 1 h, 3 h, 6 h and 24 h. At each incubation time, treated and control skin samples were processed for ultrastructural analysis.

**Figure 6:**
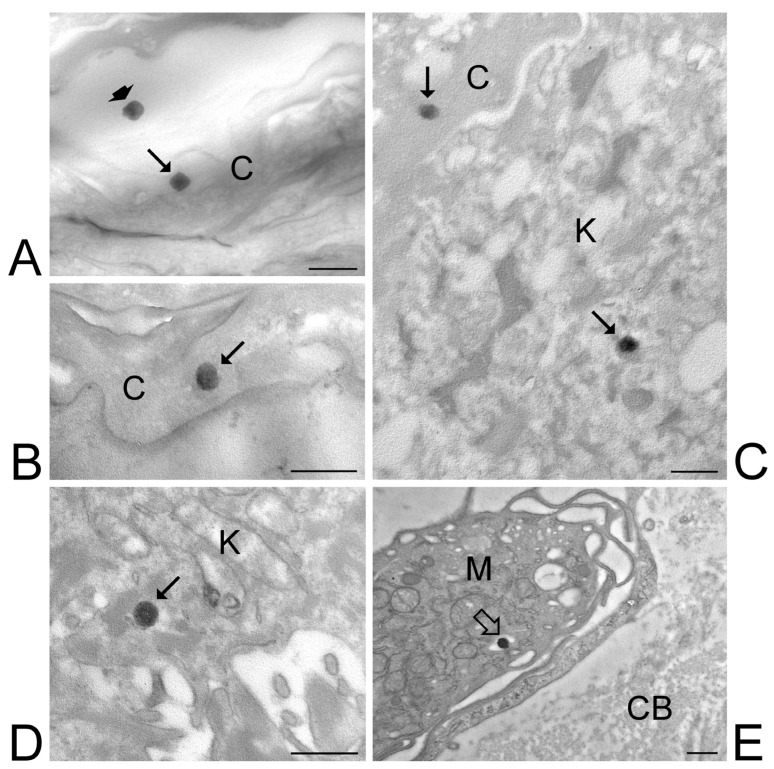
ETHO_WI_. TEM images of ETHO_WI_ in the skin. (**A**,**B**) After 1-h incubation, some ETHO_WI_ (arrows) are present inside corneocytes of the stratum corneum, while another ETHO_WI_ (arrowhead) occurs in the extracellular interstices. (**C**) After 3-h incubation, ETHO_WI_ (arrows) occur both inside corneocytes and keratinocytes of the stratum spinosum (**C**) and stratum basale (**D**). (**E**) After 6-h incubation, an ETHO_WI_ (open arrow) has been phagocyted by a macrophage in the upper papillary dermis. Corneocytes, C; keratinocyte, K; macrophage, M; collagen bundles, CB. Bars: 500 nm.

**Figure 7:**
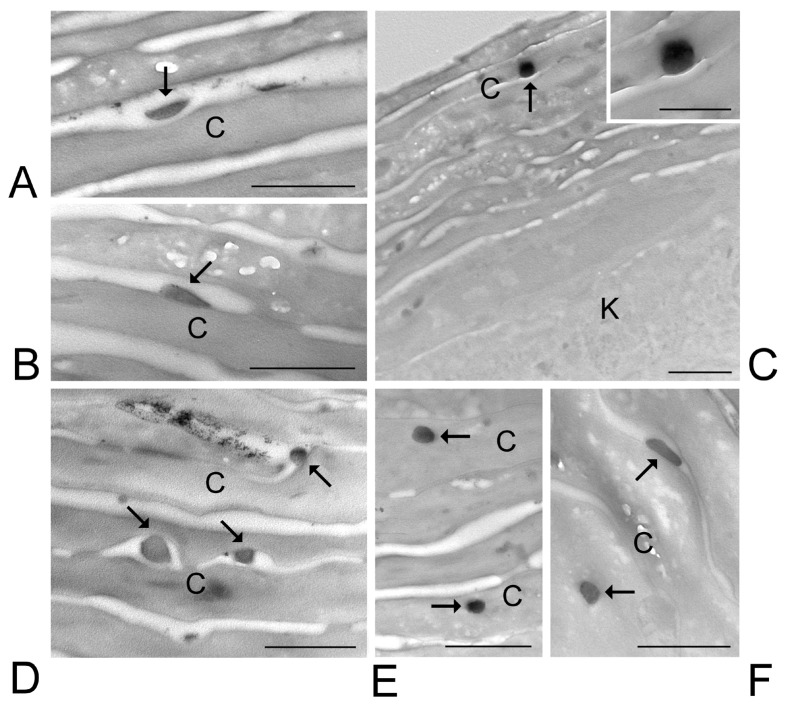
ETHO_EI_. TEM images of ETHO_EI_ in the skin. (**A**–**C**) After 1-h incubation, ETHO_EI_ (arrows) occurs (**A**) in the extracellular interstices of the *stratum corneum*, (**B**) adhering to the surface of corneocytes or (C and inset) into corneocytes. (**D**,**E**) After 3-h incubation, ETHO_EI_ (arrows) occur both (**D**) in the intracellular space of the *stratum corneum*, also adhering to the surface of corneocytes, and (**E**) inside corneocytes. (**F**) After 6-h incubation, ETHO_EI_ (arrows) occurs in the *stratum corneum*, both in the intracellular space and inside corneocytes. Corneocytes, C; keratinocyte, K. Bars: 500 nm; inset in C, 250 nm.

**Figure 8:**
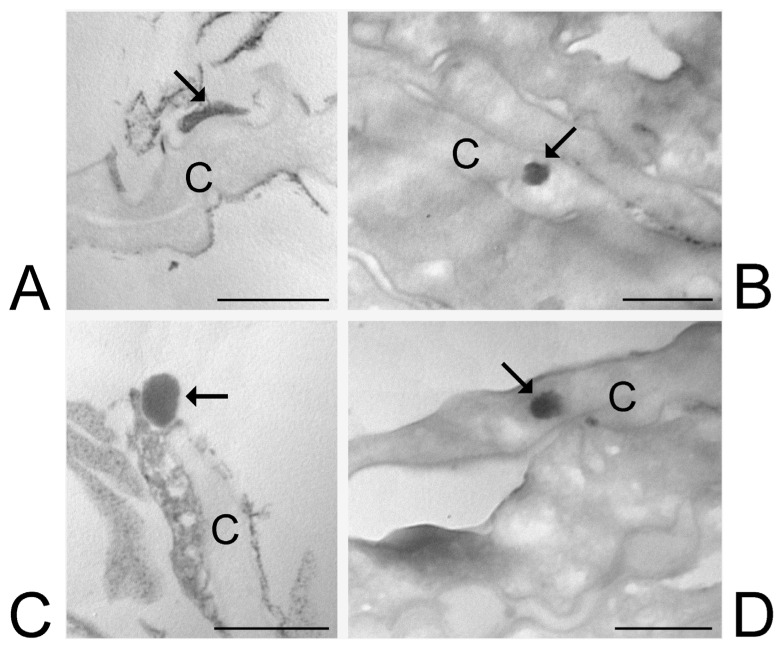
LIPO_EI_. TEM micrographs of LIPO_EI_ in the skin. Both after 1-h (**A**,**B**) and 3-h (**C**,**D**) incubation, LIPO_EI_ (arrows) were found in the *stratum corneum*, (**A**,**C**) adhering to the surface of corneocytes or (**B**,**D**) internalized into corneocytes. Corneocytes, C. Bars: 500 nm.

At TEM, ETHO_WI_, ETHO_EI_ and LIPO_EI_ were detectable in the skin components as round or ovoid electron-dense structures (Figure 6, Figure 7 and Figure 8).

After 1 h and 3 h incubation, numerous ETHO_WI_ were found among or inside corneocytes of the stratum corneum. (Figure 6A–C). Some ETHO_WI_ were also found inside keratinocytes, from the stratum spinosum to the stratum basale, although in smaller amounts compared to corneocytes (Figure 6C,D). After 6 h incubation, rare ETHO_WI_ were observed in the upper papillary dermis underlying the epidermis; in particular, ETHO_WI_ were found to be internalized into macrophages (Figure 6D). After 24 h incubation, no ETHO_WI_ was visible in any skin layer. The mean diameter of ETHO_WI_ as measured at TEM was 207.46 ± 29.07 nm.

Many ETHO_EI_ were observed after 1 h in the *stratum corneum*, both in the extracellular interstices and adhering to the surface of corneocytes (Figure 7A,B), while their occurrence inside the corneocytes was less frequent (Figure 7C). After 3 h, ETHO_EI_ were numerous in the extracellular space and inside corneocytes of the *stratum corneum* (Figure 7D,E). After 6 h, a few ETHO_EI_ were still visible at the corneocyte surface or inside them (Figure 7F). Notably, many ETHO_EI_ occurring between corneocytes showed a flattened shape. ETHO_EI_ was never observed inside keratinocytes or in the dermis. After 24 h incubation, no ETHO_EI_ was found in any skin layer. The mean diameter of ETHO_EI_ as measured at TEM was 128.92 ± 19.31 nm.

LIPO_EI_ were observed in a limited number in the *stratum corneum*, either into corneocytes or adhering to their surface, after 1 h and 3 h incubation (Figure 8). LIPO_EI_ occurring in the intercellular interstices frequently appeared as flattened (Figure 8A). LIPO_EI_ was never observed in keratinocytes or the dermis. No LIPO_EI_ was found in any skin layer after 6 h incubation. The mean diameter of LIPO_EI_ as measured at TEM was 148.15 ± 22.98 nm.

No structural alteration of cellular or extracellular skin components was observed after treatment with any nanovesicles compared to controls.

## 4. Discussion

The formulation section of this study enabled us to compare different kinds of methods for nanovesicle preparation, namely microfluidics and solvent injections. Both LIPO and ETHO were constituted of PC, ethanol, and water. Beyond its self-assembling characteristics, PC is particularly well-suited for skin administration due to its affinity with *stratum corneum* lipid composition. While PC concentration was the same in LIPO and ETHO, ethanol amount was tripled in the case of ETHO with respect to LIPO. Some authors report the need to remove alcohol from vesicles produced by microfluidics or ethanol injection since its incomplete removal could influence the stability of vesicles, leading to aggregation phenomena [53,54]. Nevertheless, in the present study, apart from LIPO_WI_ showing separation phenomena and mean size increase, ETHO_EI_, ETHO_WI_, and LIPO_EI_ displayed strong size stability, while ETHO_MF_ and LIPO_MF_ underwent a moderate size increase after 3 months. Ethanol function is crucial for PC solubilization and maintenance of nanovesicle stability while promoting their penetration through the skin. Moreover, as a general trend, the higher amount of ethanol in ETHO led to a slight reduction of mean diameter with respect to LIPO. Importantly, as previously demonstrated, the 30% *w*/*w* amount of ethanol in ETHO is considered safe for skin administration, resulting in no irritation on the human skin.

Overall, in the present research, the bulk solvent injection method was determined to be more advantageous than microfluidics, primarily due to its higher yield. Indeed, both in the case of water injection and ethanol injection, the bulk method led to 5 mL of LIPO or ETHO in 30 min. Achieving comparable nanovesicular systems by microfluidics requires a TFR, leading to a significantly smaller volume. Namely, the production volume of LIPO_MF_ and ETHO_MF_ is 3 or 1.85-fold smaller with respect to the bulk solvent injection (i.e., 1650 or 2700 µL in 30 min for LIPO_MF_ and ETHO_MF_). However, microfluidic devices can be employed in parallel by operating many microfluidic channels, achieving scalability and reproducibility in view of the industrial production of LIPO and ETHO [55].

The differences in size distribution and morphology between the nanovesicular systems produced by the different methods can be justified by examining the events during the mixing of the two phases. In the case of microfluidics, the interaction between LP and AP hydrodynamically focuses the lipid-containing stream into a narrow, rectangular sheet [54]. The vesicle formation is a result of reciprocal diffusion occurring between the lipid and water phases. This diffusion process establishes a controlled gradient of ethanol and water at their shared liquid interface. As ethanol (carrying the dissolved lipids) diffuses into the water and water concurrently enters the lipid phase, the ethanol concentration decreases until it falls below the solubility limit of the lipids. This reciprocal diffusion subsequently initiates vesicle formation through a self-assembly mechanism [56]. Consequently, the dimensional and structural attributes of the resulting LIPO_MF_ or ETHO_MF_ are strongly influenced and regulated by the concentration gradients present at the liquid interface between the solvent (ethanol) and non-solvent (water) phases, which are, in turn, determined by the focused stream’s geometric properties. Importantly, the size of this focused stream can be adjusted by modifying TFR and FRR [57].

In the case of the bulk solvent injection methods, the key mechanism is the mutual diffusion of ethanol and water at the interface, which compels PC units to associate into bilayers that subsequently fold into vesicles. The spontaneous formation of vesicles through PC self-assembly at the ethanol-water interface is a well-established phenomenon driven by the mixing process.

Regarding the bulk solvent injection method, a significant difference (approximately 100 nm) is observed in the Z-average values obtained for ETHO prepared using the different methods. Precisely, as the formulations are identical in composition, dimensional variations must be attributed to the preparation method. It can, therefore, be deduced that simply reversing the way the two phases are mixed induces significant changes in terms of size. Indeed, in the water injection method, the addition of AP into the LP promotes a thicker diffusion layer and a more gradual vesiculation process, which favors the formation of larger multilamellar structures. Conversely, in the case of the ethanol injection method, the rapid disappearance of the organic solvent leads to a thinner diffusion layer [46] since the swift solvent depletion accelerates the closure of the bilayer, resulting in smaller unilamellar vesicles. Noteworthily, the vesicle mean diameter measured by PCS agreed with Cryo-EM and hyperspectral dark microscopy observations, while the ultrastructures of nanovesicles measured by SAXS are in good agreement with Cryo-EM findings.

Since both size and lamellarity of the vesicles can strongly influence their delivery process and their passage through the skin [28,58], we decided to select the unilamellar nanovesicular systems obtained by ethanol injection method (ETHO_EI_ and LIPO_EI_), characterized by the smallest size, and to compare their behavior under skin administration with the larger multilamellar ETHO_WI_, previously investigated by our research group [36].

Thus, to shed light on the vesicle transdermal fate investigating the impact of size and lamellarity, ex-vivo distribution studies were conducted on ETHO_WI_, ETHO_EI_ and LIPO_EI_.

ETHO_WI_ were previously demonstrated to penetrate the skin crossing the epidermis in 3 h, while their presence in the dermis was quite rare [36]. In the present work, we provide original evidence that ETHO_WI_ reaching the papillary dermis undergoes phagocytosis by the resident macrophages. The administered ETHO_WI_ -or at least many of them- are therefore able to preserve their morphological integrity during the passage through the epidermis strata, being detectable until they reach the dermis, where they disappear because they are removed by macrophages. This is consistent with a previous in vitro study [39] where ETHO_WI_ internalized in keratinocytes was demonstrated to be only partially degraded by intracellular phospholipases, remaining easily recognizable at TEM until 24 h from administration. It is evident that their degradation is more rapid in the epidermis than in cultured cells: ETHO_WI_ penetrates the *stratum corneum* by sliding into the extracellular space and then enters corneocytes and keratinocytes, undergoing lytic degradation. Notably, keratinocytes are linked by numerous desmosomes, whose integrity is guaranteed using the bioreactor [38], thus making the extracellular passage of ETHO_WI_. The transcellular way is, therefore, the most probable route followed by ETHO_WI_ to penetrate the epidermis, according to the already proven ability of these nanovesicles to easily cross the cell membrane [39]. The multilamellar structure of ETHO_WI_ could be responsible for their fate. Indeed, they probably possess a higher affinity with cell membranes with respect to ETHO_EI_ and LIPO_EI_, characterized by unilamellar vesicles. Indeed, vesicle lamellarity, a variable determined by the preparation method, profoundly influences a range of vesicle behaviors, including encapsulation capacity, the rate at which encapsulated substances escape, the regulation of drug release, and penetration capabilities. Additionally, lamellarity significantly affects the intracellular handling of drugs carried by vesicle post-cellular uptake [59].

ETHO_EI_ exhibited a lower capability to penetrate the skin as intact nanovesicles compared to ETHO_WI_. In fact, ETHO_EI_ has never been observed in keratinocytes, even in the upper layer (*stratum spinosum*). Their presence remained confined to the *stratum corneum*, where they occur both in the intercellular interstices and inside corneocytes. It may be, therefore, hypothesized that their capability to cross cell membranes is limited in comparison to ETHO_WI_. On the other hand, ETHO_EI_ demonstrated resistance to lytic degradation like ETHO_WI_ since they were morphologically recognizable until 6 h from their administration. Notably, ETHO_EI_ occurring in the interstices between corneocytes often showed a flattened shape, suggesting that their unilamellar structure could confer a higher flexibility to the vesicles with respect to the multilamellar ETHO_WI_. Thus, it can be assumed that the ETHO_EI_ can deform during passage through the skin while maintaining its structure intact.

Moreover, LIPO_EI_ demonstrated scarce penetration capability and high degradability. In fact, they were found in low numbers only in the *stratum corneum* (both inside corneocytes and in the interstices between them) and only until 3 h from administration. As observed for ETHO_EI_, many LIPO_EI_ showed flattened shapes in the intercellular spaces, suggesting that their unilamellar structure can confer flexibility to the vesicles. Notably, the poor penetration of LIPO_EI_ can be attributed to the lower amount of ethanol in their composition, being 3-fold lower with respect to ETHO_WI_ and ETHO_EI_. In this respect, the role of ethanol as a penetration enhancer appears to be crucial in promoting the vesicle passage through the skin.

## 5. Conclusions

The presented findings confirm that both the modality of vesicle preparation and the amount of ethanol affect the mean size and inner structural organization of LIPO and ETHO. Particularly, PCS demonstrated that the ethanol injection method enabled the obtain of nanovesicles with Z-Average mean diameters of 130–145 nm, while the water injection and microfluidic methods resulted in vesicles measuring 225–235 nm mean diameters. LIPO_WI_ underwent vesicle aggregation and phase separation phenomena. The stability study evidenced that ETHO_EI_, ETHO_WI_, and LIPO_EI_ maintained almost unvaried Z-Average mean diameters over 3 months. Cryo-EM, SAXS and hyperspectral dark-field microscopy shed light on the nanocarrier ultrastructure, characterized by unilamellar bi-layered vesicles in the case of ETHO_EI_ and LIPO_EI_ and multilamellar bi-layered vesicles in the case of ETHO_WI_.

Remarkably, the lamellarity of the vesicles seems to influence their fate, resulting in different degradation times and a deeper skin interaction in the case of multilamellar vesicles. Particularly, the ETHO_WI_ uptake in keratinocytes suggests the vesicle potential to deliver the loaded drug directly into the cells. In addition, the deeper permeation of ETHO with respect to LIPO corroborates the penetration enhancer role of ethanol, confirming the transdermal potential of both ETHO_WI_ and ETHO_EI_. Further studies will be performed to better investigate the skin penetration mechanisms of the vesicles. Moreover, investigations conducted on inflamed skin biopsies will shed light on the possibility of ETHO loaded with anti-inflammatory agents to counteract the inflammatory status induced by exogenous stimuli.

## Figures and Tables

**Table 1:** Preparation parameters and compositions of nanovesicular systems.

Nanovesicular System	TFR ^a^ (μL/min)	FRR ^b^	F_AP_ ^c^ (μL/min)	F_LP_ ^d^ (μL/min)	PC ^e^ (%, *w*/*w*)	Ethanol (%, *w*/*w*)	Water (%, *w*/*w*)
LIPO_MF_	55	10:1	25 + 25	5	0.9	9.1	90
ETHO_MF_	90	2:1	30 + 30	30	0.9	29.1	70
LIPO_WI_	-	-	1500	-	0.9	9.1	90
ETHO_WI_	-	-	1500	-	0.9	29.1	70
LIPO_EI_	-	-	-	1500	0.9	9.1	90
ETHO_EI_	-	-	-	1500	0.9	29.1	70

^a^: Total Flow Rate (F_AP_ + F_LP_); ^b^: Flow Rate Ratio (F_AP_/F_LP_) volumetric aqueous phase/lipid phase ratio; ^c^: Aqueous Phase Flow; ^d^: Lipid Phase Flow; ^e^: soy phosphatidylcholine.

**Table 2:** Size distribution parameters of nanovesicular systems.

Nanovesicular System	Z-Average (nm) ± s.d.	Dispersity Index ± s.d.	Typical Intensity Distribution (nm)	Macroscopic Aspect
LIPO_MF_	234 ± 10	0.24 ± 0.01	233.02 (91%)	milky, homogeneous
ETHO_MF_	226 ± 7	0.26 ± 0.03	238.21 (90%)	milky, homogeneous
LIPO_WI_	235 ± 14	0.27 ± 0.02	226.61 (97%)	phase separation
ETHO_WI_	225 ± 9	0.20 ± 0.01	225.11 (95%)	milky, homogeneous
LIPO_EI_	144 ± 3	0.17 ± 0.01	143.21 (98%)	milky, homogeneous
ETHO_EI_	130 ± 13	0.15 ± 0.02	138.40 (100%)	milky, homogeneous

**Table 3:** Main parameters resulting from SAXS data analysis. Fixed parameters were *ρ*_1_ = 370 e/nm^3^, *ρ*_2_ = 320 e/nm^3^, *ρ*_3_ = 300 e/nm^3^,*ρ*_0_ = 330 e/nm^3^ and *ρ_EtH_* = 285 e/nm^3^.

	LIPO_EI_	ETHO_WI_	ETHO_EI_
*R*_1_ (nm)	0.49 ± 0.07	0.66 ± 0.04	0.64 ± 0.02
*R*_2_ (nm)	1.19 ± 0.02	1.03 ± 0.06	1.04 ± 0.02
*R*_3_ (nm)	0.10 ± 0.02	0.16 ± 0.05	0.14 ± 0.02
*N*	6 ± 2	5 ± 2	2 ± 4
*c* (nm)	17 ± 10	14 ± 1	35 ± 9
*g_c_*	0.9 ± 0.1	0.14 ± 0.01	0.5 ± 0.2
*R*_0_ (nm)	500 ± 100	488 ± 85	500 ± 90
ξ*_Ro_*	0.1 ± 0:2	0.01 ± 0.06	0.0 ± 0.2

## Data Availability

Data will be made available on request.

## Abbreviations

The following abbreviations are used in this manuscript:

LIPO	Liposomes
ETHO	Ethosomes
WI	Water injection
EI	Ethanol injection
PC	Phosphatidylcholine
PCS	Photon Correlation Spectroscopy
Cryo-EM	Cryogenic Electron Microscopy
SAXS	Small Angle X-Ray Scattering
TEM	Transmission Electron Microscopy
AP	Aqueous Phase
LP	Lipid Phase
TFR	Total Flow Rate
FRR	Flow Rate Ratio
